# In-Vitro Decidualization With Different Progesterone Concentration: Development of a Hormone-Responsive 3D Endometrial Tissue Using Rat Endometrial Tissues

**DOI:** 10.7759/cureus.49613

**Published:** 2023-11-28

**Authors:** Chihiro Toma, Goro Kuramoto, Jun Homma, Katsuhisa Sakaguchi, Tatsuya Shimizu

**Affiliations:** 1 Department of Obstetrics and Gynecology, Tokyo Women's Medical University, Tokyo, JPN; 2 Department of Clinical Regenerative Medicine, The Center for Advanced Reproductive Medicine, Fujita Medical Innovation Center Tokyo, Tokyo, JPN; 3 Institute of Advanced Biomedical Engineering and Science, TWIns, Tokyo Women's Medical University, Tokyo, JPN; 4 Department of Medical Engineering, Faculty of Science and Engineering, Tokyo City University, Tokyo, JPN

**Keywords:** decidualization, three-dimensional tissue, progesterone, cell sheet engineering, endometrial stromal cell, endometrial epithelial cell

## Abstract

Infertility in women is associated with various uterine and ovarian disorders. Treatment strategies for infertility can range from medications to embryo implantation through assisted reproductive technology (ART). ART has enabled considerable progress; however, there is currently no treatment to replace the endometrium itself. Decidualization requires a complex interaction between endometrial tissue and estrogen and progesterone. We aimed to create a three-dimensional endometrial-like tissue model using in-vitro cell sheet engineering with rat endometrium, and culture cells at different progesterone concentrations to mimic local concentrations. Histological and morphological changes revealed that development of the endometrial-like tissue was not proportional to progesterone concentrations in terms of thickness, number of endometrial glands, or area fraction of intimal glands. These results suggest that decidualization may not be commensurate with the local endometrial progesterone concentration. Notably, the number of endometrial glands increased in the high concentration group and compaction occurred, indicating that the endometrial conditions in the high concentration group may be most conducive to increase pregnancy rates. These findings suggest that there may be an “optimal progesterone concentration” for decidualization, application of which may lead to new strategies for improving pregnancy rates in women with infertility.

## Introduction

Various medical treatments have been adopted for infertility, and the number of patients undergoing assisted reproductive technology (ART) is increasing [1]. ART has advanced considerably, with technologies such as in vitro fertilization (IVF) and intracytoplasmic sperm injection (ICSI). These new modalities have contributed to an increase in pregnancy rates.

However, physicians have experienced that in some patients there is repeated implantation failure even when good quality fertilized eggs are transplanted multiple times. To improve the pregnancy rate, together with requirement of good fertilized egg and healthy endometrium, optimal interaction between the egg and the endometrium is also required. Previous studies have revealed the cross-talk between fertilized eggs and the endometrium, and several experimental systems have been reported to analyze the relationship between fertilized eggs and the endometrium [2-4]. The study of cross-talk between the fertilized egg and the endometrium requires an endometrial model that is as close to living tissue as possible. To this end, in-vitro endometrial models have been created using stromal cells coated with collagen or Matrigel. Subsequent seeding of epithelial cells on top of the layers generated a three-dimensional endometrial tissue, which was used to observe embryo implantation [5-6]. In addition, stromal cells were placed in a fibrin-agarose gel and endometrial tissue was created by seeding epithelial cells onto the gel. Another study showed that implantation of a choriocarcinoma cell line on top of the tissue can mimic implantation of fertilized eggs [7]. However, tissues created in these studies did not exhibit the tight cell-cell interaction that exists in living organisms.

Furthermore, the diameter of a fertilized rat egg is approximately 50 µm, while that of a fertilized human egg is approximately 140 µm. To observe the implantation and invasion of fertilized eggs, it is necessary to construct a tissue model with a thickness commensurate with the size of the fertilized egg. Potential solution to these problems is creating the 3D endometrium tissue, which can be explained by cell sheet engineering, which is a regenerative medicine technology that enables the collection of cells as sheets, and three-dimensional tissues can be fabricated by layering the cell sheets [8]. Shimizu et al. reported that cardiomyocyte cell sheet layering were able to construct thick tissues of up to 80 μm without generation of vascular networks [9]. Sakaguchi et al. and Sekine et al. succeeded in constructing three-dimensional myocardial tissue composed only of cells with vascular structures, by layering cell sheets from rat cardiomyocytes in vitro [10-11]. Cell sheet engineering has been applied to various organs such as the heart, cornea, and liver. These studies have demonstrated that cell sheets can replace tissues and promote tissue repair via the paracrine effect [12-14]. Cell sheet engineering has been applied clinically and has contributed to the development of regenerative therapy [15-16].

Endometrial sheets using cell sheet engineering are under investigation in vitro. This technique was used to create cell sheets of endometrial epithelial and stromal cells from the endometrium removed from rats in vitro, and transplantation into adult female rats with uterine endometrial damage examined [17]. In addition, embryo implantation and normal pregnancy were confirmed when the cell sheets were replaced at the endometrial defect site. The result suggested that the created in vitro cell sheets could replace the endometrium in living organisms [18]. The created sheets were also observed in vitro under the influence of various in vivo factors, such as the hormones of the transplanted rats. The changes in the cell sheets under the influence of different hormone concentrations and altered environments have not been investigated.

Progesterone and estrogen induce and regulate the decidualization of human endometrial stromal cells in a time-dependent manner. Progesterone is necessary for implantation and invasion of the fertilized egg, as well as for maintenance of pregnancy. However, human serum progesterone levels are highly variable, and there is no marker to determine the optimal local concentration for embryo implantation. Furthermore, species differences and ethical issues can influence experimental systems and clinical applications [19]. Currently, there is no consensus on the optimal progesterone concentration for embryo transfer.

In this study, endometrial sheets were prepared from rat endometrial tissue using cell sheet engineering and layered sheets to create three-dimensional endometrial-like tissue with a thickness that allows observation of fertilized egg implantation and invasion. The 3D tissue was cultured in vitro at different progesterone concentrations and the histological and morphological changes of decidualization were observed to determine the appropriate local progesterone concentration for implantation of the fertilized egg.

## Materials and methods

Animals

Female scl: SD rats (Sankyo Labo Service Corporation, Inc., Tokyo, Japan) aged 3-4 weeks were used. All rats used in the experiments were housed in an environmentally controlled room. They received food and water ad libitum under a 12-h light/dark cycle and were allowed to move freely in their cages. All animal experiments were conducted in accordance with the guidelines established by the Institutional Animal Committee of Tokyo Women's Medical University (Approval No. AE20-122, 2020).

Harvesting endometrial epithelial cell sheets and stromal cell sheets

**Figure 1:**
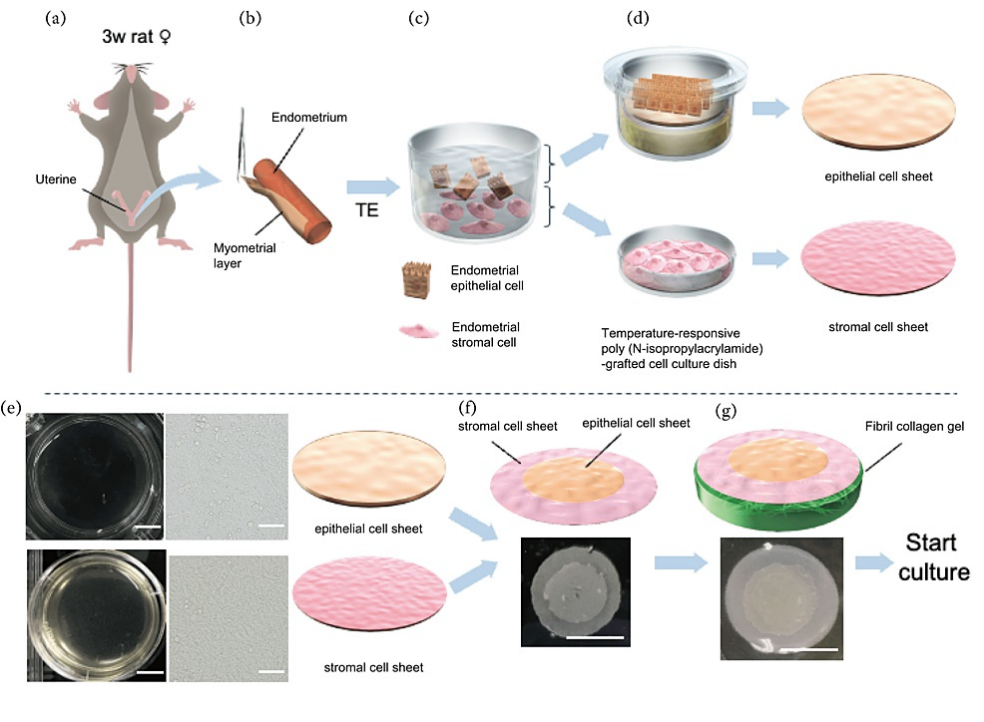
How to make endometrial like tissue (a)〜(d)How to make endometrial epithelial cells and stromal cells sheet using cell sheet engineering. (e) Four days after culturing, an endometrial epithelial sheet and stromal cell sheet on the culture dish. Both dishes were confluent. (scale bar 50 μm) (f) Layered epithelial sheet and stromal cell sheet cell sheet (scale bar 1 cm) (g) After layered sheets, placed them at the collagen gel. (scale bar 1 cm)

Endometrial cell sheets were prepared as previously reported [17-18]. Briefly, the endometrial layer was removed from the uterus of 3-4-week-old rats (Figures 1a-1b).

The collected endometrial tissue was treated with 0.5% trypsin-EDTA, and the treated endometrial tissue was isolated using 100 and 40 mm pore-size filters. After centrifugation, the suspended cells were seeded on 100 mm dishes and incubated with DMEM/Ham's F-12 containing L-Gln, sodium pyruvate, and HEPES, without phenol red (15177-15, Nacalai Tesque, Kyoto, Japan), 1% penicillin-streptomycin solution (168-23191, FUJIFILM Wako, Osaka, Japan), and 10% fetal bovine serum (FBS; 175012, NICHIREI BIOSCIENCES INC, Tokyo, Japan). After incubating for 4 h, the supernatant fluid in the 100 mm dishes was collected and used as endometrial epithelial cells. The remaining attached cells on the bottom of the dishes were collected by trypsin treatment as the endometrial stromal cells. The 100 mm dishes were washed with D-PBS (14249-24, Nacalai Tesque) 5-6 times and the endometrial epithelial cells that were attached to the walls of the dishes were collected (Figure 1c).

As a basement medium, advanced DMEM/F12 (12634-010, Thermo Fisher Scientific, MA, USA) with 2 mM L-Glutamine solution (G7513, Sigma Aldrich MO, USA), 8nM β-Estradiol (E8875, Sigma), 4 ng/mL progesterone (P8783, Sigma), 25 μM N-acetyl-L-cysteine (017-05131, FUJIFILM Wako), 1% ITS -X (51500056, Thermo Fisher Scientific), and 1% penicillin-streptomycin was prepared. Endometrial epithelial cells were seeded on a temperature-responsive poly(N-isopropylacrylamide)-grafted cell culture insert (CS6301, CellSeed, Tokyo, Japan) at 6.0 × 106 cells per insert precoated with FBS for 4 days with endometrial epithelial culture medium, as previously described [17-18]. Attached cells, endometrial stromal cells, were seeded on a temperature-responsive cell culture dish (CS3007, CellSeed) at 10.0 × 106 cells per dish precoated with Easy iMatrix-511 (892018, Takara Bio Inc., Shiga, Japan) for 4 days with basement medium added with 10% embryonic stem-cell FBS (1043900, Thermo Fisher Scientific) and 15% KnockOut™ Serum Replacement (10828010, Thermo Fisher Scientific) (Figure 1d). Four days after culturing, an epithelial cell sheet and a stromal cell sheet were harvested by reducing the temperature to 20°C in a 5% CO2 incubator for 30 min (Figure 1e).

Creating an in-vitro three-dimensional endometrium tissue system by layering cell sheets

A floating endometrial stromal cell sheet was placed on a culture dish by removing the culture medium, and leaving on the dish for 30 min at 37°C. Subsequently, the floating endometrial epithelial cell sheet was transferred onto the stromal cell sheet using a plastic sheet and the layered cell sheet was cultured for 5 h at 37°C (Figure 1f). A fibrin-collagen gel base was created as described in previous studies [8,20]. Briefly, to make the gel base, 2.11％ medium 199 (11825015, Thermo Fisher Scientific), was mixed with 1.59 mM D-(+)-glucose (16805-64, Nacalai Tesque), 0.144 mM L-(+)glutamine (076-00521, FUJIFILM Wako), 15.3 mM NaCl (191-01665, FUJIFILM Wako), 0.118 mM NaH2PO4-2H2O (196-13685, FUJIFILM Wako), 0.107 mM MgSO4-7H2O (137-00402, FUJIFILM Wako), 0.155 mM KCL (169-03542, FUJIFILM Wako), 0.545 mM CaCl2 (039-00475, FUJIFILM Wako), 0.150 mM NaHCO3 (FUJIFILM Wako), 0.05 mM HEPES (342-01375, DOJINDO LABORATORIES, Kumamoto, Japan), 0.625 U/mL thrombin solution (T4648, Sigma), 5 mg/mL fibrinogen (F8630, Sigma), 2 mg/mL native collagen acidic (IAC-50, KOKEN CO., LTD., Tokyo, Japan) and placed in a donut-shaped silicone mold (diameter: 1.2 cm) at the center of a 60-mm dish. After 4 h of incubation for gel setting, the mold was removed from the dish and the fibrin-collagen gel was attached to the dish. A layered cell sheet was placed at the center of the gel and expanded by removing the medium. The expanded sheet was incubated to attach to the gel for 1-2 h at 37°C (Figure 1g).

Progesterone stimulation on three-dimensional rat endometrium tissue system

After attachment to the gel base, the sheets and gel base were placed on a cell culture insert (353092; CORNING, AZ, USA). The 3D endometrial tissue system was then incubated in basement medium containing 20% embryonic stem cell FBS and progesterone at concentrations of 4, 200, 1000, and 2000 ng/mL. After initiating culture, the 3D endometrial tissue was monitored daily using optical coherence tomography (OCT). After 3 days of incubation, half of the 3D tissue was fixed with 4% paraformaldehyde for histological analysis, and the other half was frozen with liquid nitrogen for gene expression analysis.

Histology and immunohistochemistry

The paraffin-embedded samples were separated equally into five small blocks including the center of the tissue. Each section was stained with hematoxylin and eosin using conventional methods.

For immunostaining, the sections were activated by warm bath treatment with Dako Target Retrieval Solution, citrate pH 6 (S2369, Agilent Technologies, CA, USA), and blocked with Blocking One Histo (06349-64, Nacalai Tesque) using a pressure cooker. The primary antibodies used for immunohistochemical staining were anti-cytokeratin 18 [C-04]; ab668; Abcam, Cambridge, UK), anti-vimentin [VI-10]; ab20346; Abcam), anti-progesterone receptor [SP42] (ab101688; Abcam), and anti-estrogen receptor beta (ab3576; Abcam). The secondary antibodies were goat anti-rabbit IgG antibody, Alexa Fluor™ 488 (A11008, Thermo Fisher Science) and goat anti-mouse IgG antibody, Alexa Fluor™ 568 (A11031, Thermo Fisher Science). After the second secondary antibody response, ProLong™ Gold Antifade Mountant with DAPI (P36935, ThermoFisher) was used for nuclear staining and enclosure.

Measurement of the 3D endometrium thickness and the number of endometrial glands after progesterone stimulation

The prepared sections were observed under upright and fluorescence microscopes, and the images were analyzed using ImageJ software (Wayne Rasband et al., National Institutes of Health). The thickness of the endometrial tissue was measured using the ImageJ software with hematoxylin-eosin-stained specimens. The horizontal length was obtained by connecting the centers of the longitudinal widths at multiple locations in the tissue using a line. The total area was divided by the length of the horizontal axis to obtain the average length of the vertical axis, which was used as the thickness. The endometrial glands were counted as the CK18 positive cells creating a gland structure with immunohistochemical staining under a fluorescence microscope. The area of the endometrial glands was also measured and calculated as a ratio of the whole cell sheet area using the ImageJ software.

Gene expression assay by quantitative real-time reverse transcription-polymerase chain reaction

After 3 days of incubation, all 3D endometrial samples were frozen in liquid nitrogen. The cell sheets were immediately frozen as a control. RNA extraction was performed using the RNeasy Fibrous Tissue Mini Kit (74704; QIAGEN, Hilden, Germany), following the recommended protocol. A NanoDrop 2000/2000c ultraviolet-visible spectrophotometer (ND-2000C, Thermo Fisher Scientific) was used to measure the extracted RNA concentrations. The extracted RNA was amplified using a ProFlex PCR System (Thermo Fisher Scientific) following the recommended protocol using a High-Capacity cDNA Reverse Transcription Kit (4368814, Thermo Fisher Scientific) for synthesize DNA.

Target primers were added to the amplified DNA (cDNA) and qPCR was performed using a ViiATM 7Real-Time PCR System (4453723; Thermo Fisher Scientific). Insulin-like growth factor-binding protein 1 (IGFBP1, Assay ID Rn00565713_m1, 4331182, Thermo Fisher Scientific) was used as the target primer, and glyceraldehyde-3-phosphate dehydrogenase (GAPDH, Assay ID Rn01775763_g1, 4331182, Thermo Fisher Scientific) was used as the control. The average ΔΔCT value was calculated with the obtained ΔCT, and the average relative expression of each progesterone concentration was determined as an average expression of the control group which was set to 1.

Statistical analysis

The thickness of the endometrial cell sheet, the number of the endometrial glands, and the area ratio of endometrial glands in all the progesterone concentration groups were confirmed to be non-normally distributed by the Shapiro-Wilk test with JMP® Pro 16 (SAS Institute Inc., NC, US).

Subsequently, the Kruskal-Wallis test was performed as a non-parametric test. For multiple comparisons, the Steel-Dwass test was performed. Outliers were excluded using Huber’s M test. In all tests, p≤ 0.05 and p ≤0.01 were considered statistically significant.

## Results

**Figure 2:**
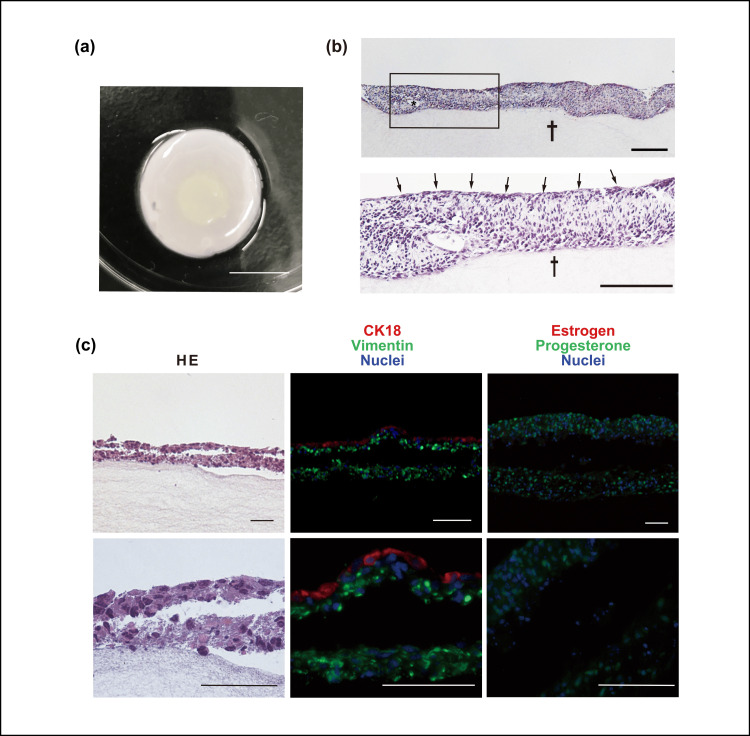
Histological examination in layered epithelial sheet and stromal cell sheet (a) Layered epithelial sheet and stromal cell sheet on the gel. (scale bar 1 cm) (b) Layered sheets with hematoxylin-eosin stain. The superficial cells had a cuboidal shape and one consecutive layer, similar to the original endometrial epithelium (arrows). Two-layered cell sheet on the fibrin-collagen gel base (dagger). (scale bar 100 μm) (c) Layered sheets at immunohistochemistry. (scale bar 50 μm)

Endometrial epithelial and stromal cells were collected and seeded on temperature-responsive cell dishes (Figures 1a-1d), and endometrial epithelial and stromal cell sheets were harvested from the dishes (Figure 1e). The cell sheets could be easily transferred to another location using a plastic sheet, and the epithelial cell sheet was layered on the stromal cell sheet to create the three-dimensional (3D) endometrial tissue (Figure 1f). Additionally, the layered cell sheet was transferred onto a fibrin-collagen gel base to fabricate an in vitro 3D endometrial tissue system (Figure 1g). A layered cell sheet was uniformly observed at the center of the fibrin-collagen base (Figure 2a).

**Figure 3:**
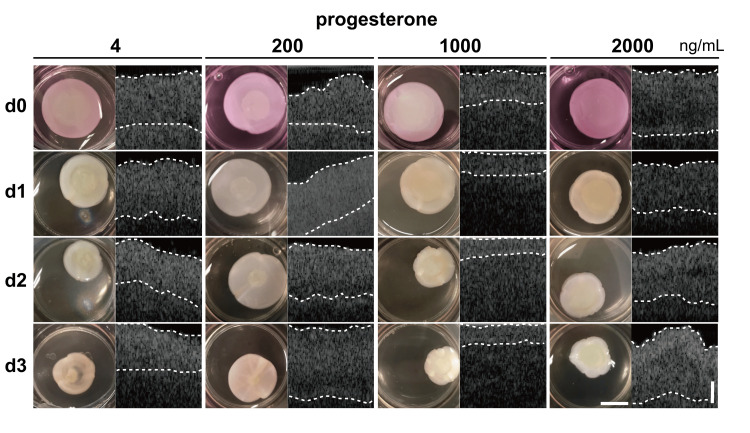
Endometrial epithelial cell sheet and stromal cell sheet at different progesterone concentrations: changes in physical and OCT. Two-layered cell sheet (dotted line) on the gel. (physical: scale bar 1 cm, OCT: scale bar 100 μm)

Hematoxylin-eosin staining revealed a two-layered cell sheet on the fibrin-collagen gel base. The upper cell sheet layer contained two cell types. The superficial cells had a cuboidal shape and one consecutive layer, similar to the original endometrial epithelium (Figure 2b arrow). The second layer beneath the cell sheet contained one type of cell that was similar to the original endometrial stromal cells. The superficial cell layer was CK18-positive and the other cells were vimentin-positive. Therefore, the layered cell sheet resembled a multilayer endometrial structure. Additionally, this tissue possessed a progesterone receptor in the wide stromal layer, which was consistent with the vimentin-positive layer. However, no estrogen-positive cells were observed in this tissue (Figure 2c). After progesterone stimulation, the tissue was maintained on the gel base for at least 3 days in all progesterone concentration groups (Figure 3).

On Day 0 after incubation, the layered cell sheets in all progesterone concentration groups adhered to the gel, and there was no floating or peeling during incubation. On Day 1, less change and shrinkage of the structure was observed in the 2000 ng/mL progesterone group (P-2000), whereas tissue contraction was observed in the 4, 200 and 1000 ng/mL progesterone concentration groups (P-4, P-200 and P-1000). On Day 2, the tissues in the P-1000 and P-2000 groups moved closer to the center of the gel base. However, the tissues in P-4 and P-200 contracted and raised up with the gel. Finally, the tissues in all groups were contracted and raised up with the gel on Day 3. However, the magnitude of the change depended on progesterone concentration, from the low-concentration group (P 4 ng/mL) to the high-concentration group (P 2000 ng/mL). The OCT system was used to evaluate the cell sheet attachment to the gel base over a period of 3 days (right side of each figure in Figure 3). On Day 0, there were no gaps between the epithelial and stromal cell sheets or between these cell sheets and the gel base. In the P-4 group, the thickness did not change from Day 0 to Day 1; however, the thicknesses on Days 2 and 3 were slightly lower than that on Day 0, whereas the P-1000 group exhibited greater thickness. In contrast, in the P-200 group, the thickness on Day 1 was greater than that on Day 0. From Days 2 to 3, the thicknesses increased, and the tissue on Days 1, 2, and 3 was thicker than on Day 0. A similar trend was observed for the P-2000 group. In the P-2000 group, the thickness scarcely changed from Day 0 to 3.

**Figure 4:**
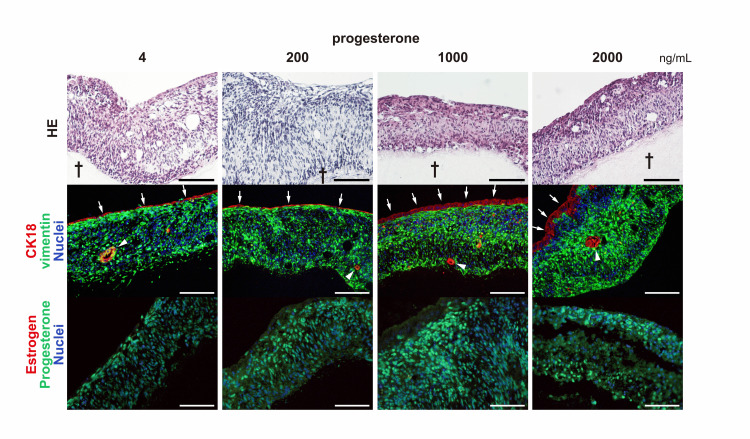
Histological and immunohistochemistry examination in endometrial epithelial cell sheet and stromal cell sheet at different progesterone concentrations) Two-layered cell sheet on the fibrin-collagen gel base (dagger). Immunohistochemical staining revealed that the superficial monolayer cells (arrows) and glandular cells (arrowheads) were CK18-positive, and the layer beneath the superficial layer was vimentin-positive in all progesterone concentration groups. (scale bar 100 μm)

We performed hematoxylin-eosin histological staining of tissue sections for each progesterone concentration (Figure 4).

Hematoxylin-eosin staining revealed a squamous cell monolayer on top of the tissue and stromal cells beneath the squamous monolayer as a thick tissue layer. Each of the monolayer squamous cells was attached tightly; the layer did not peel off from the tissue, and the entire layered cell sheet tissue was attached to the gel base. Immunohistochemical staining revealed that the superficial monolayer cells (arrows in Figure 4) and glandular cells (arrowheads in Figure 4) were CK18-positive, and the layer beneath the superficial layer was vimentin-positive in all progesterone concentration groups. In the endometrium in the living body, there are monolayer CK18 cells that provide the endometrial epithelium with a luminal structure, with endometrial glands in the stromal layer. The 3D tissue structure was very similar to the endometrial structure in the living body. Progesterone receptor-positive cells were identified in the wide layer of the 3D tissue but not in the superficial layer. No estrogen receptor-positive cells were observed in any of the progesterone concentration groups.

**Figure 5:**
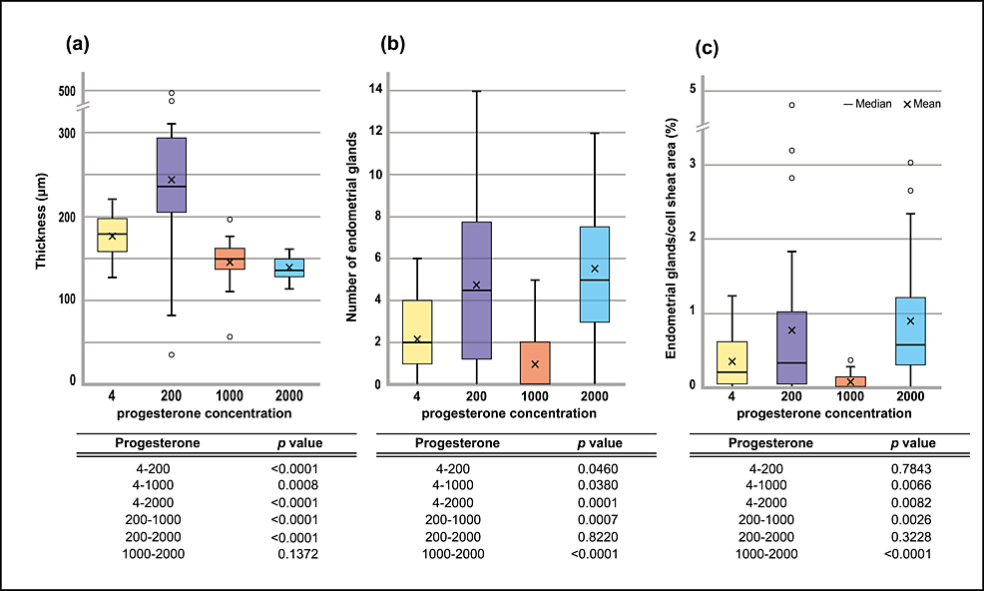
Effect of progesterone stimulation to in vitro endometrium 3D tissue system.

The median and mean (±2SD) thickness in the P-4 group were 178.46 μm, and 176.03±24.6 μm, respectively (Figure 5a).

Similarly, those in P-200, P-1000, and P-2000 groups were 235.75 mm, 257.58±140 mm, 148.48 mm, 145.51±26.2 mm and 135.71 mm, 138.97±15.8 mm, respectively. All progesterone concentration groups were non-normally distributed. Because of this result, we used a non-parametric analysis of variance test. The nonparametric test showed statistical significance (p<0.05). In the multiple comparisons, there were significant between-group differences for all comparisons except between P-1000 and -2000. P< 0.01 was achieved for comparisons between P-4 and P-200, P-4 and P-1000, P-4 and P-2000, P-200 and P-1000, and P-200 and P-2000.

The number of endometrial glands was counted and compared between groups (Figure 5b). The median and mean (±2SD) number of endometrial glands in P-4 were 2.0 and 2.2 ± 1.81, respectively. Similarly, those for the P-200, P-1000 and P-2000 groups were 4.5, 4.8±3.73; 0.0, 1.0±1.51; and 5.0, 5.6±2.97, respectively. All progesterone concentration groups were non-normally distributed. Because of this result, we used a non-parametric test for analysis of variance. The nonparametric test showed p<0.05. In multiple comparisons, there was a significant difference between all two-group-comparisons except between P-200 and P-2000. Comparisons between P-4 and P-200, P-4 and P-1000 achieved p<0.05, whereas P-4 and P-2000, P-200 and P-1000, P-1000 and P-2000 all exhibited p<0.01.

Using the same method, we examined the area fraction of intimal glands in the cell sheets (Figure 5c). The median and mean (±2SD) of the number of endometrial glands at P 4 ng/mL were 0.20, and 0.35±0.369, respectively. Similarly, they were as follows for P 200 ng/mL (0.32, 0.81±1.28), P 1000 ng/mL (0.0, 0.078±0.119), and P 2000 ng/mL (0.57, 0.90±0.820). All progesterone concentration groups were non-normally distributed. We therefore used a nonparametric test. The nonparametric test showed p<0.05. In the multiple comparisons, there was a significant between all two-group-comparisons except between P-4 and P-200, and P-200 and P-2000. P-200 and P-1000 showed p<0.05. P-4 and P-1000, P-4 and P-2000, P-1000 and P-2000 achieved p<0.01.

Considering the influence of outliers on the analysis results, the outliers were identified and excluded using Huber’s M test. The endometrial sheet thickness and area fraction of the intimal glands in the cell sheets had outliers. After excluding outliers, the non-parametric test for endometrial sheet thickness and the area fraction of endometrial glands in the cell sheets was p<0.05. In the multiple comparisons of endometrial sheet thickness, there was a significant difference between all two-group-comparisons except between P-1000 and P-2000. Comparisons between P-4 and P-200, P-4 and P-1000, P4- and P-2000, P-200 and P-1000, and P-200 and P-2000 showed p<0.01. In the same comparisons of the area fraction of intimal glands in cell sheets, there were significant differences between all two-group-comparisons except between P-4 and P-200, and P-200 and P-2000. Comparisons between P-4 and P-2000, P-200 and P-1000 achieved p<0.05. Comparisons between P-4 and P-1000, and P-1000 and P-2000 obtained p<0.01.

**Figure 6:**
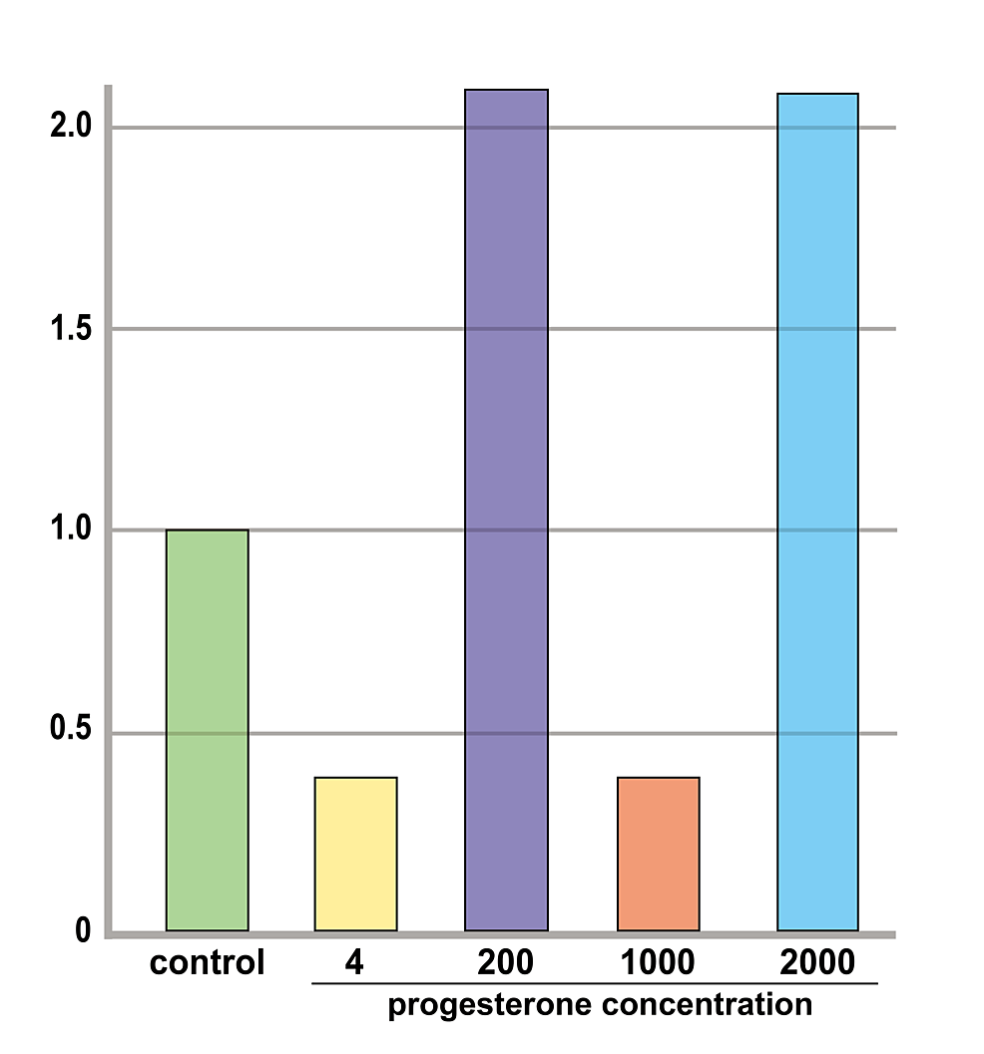
The gene expression level of IGFBP-1 by qPCR The results of qPCR analysis of IGFBP-1 for each progesterone concentration group. When the average relative expression of the control group was set to 1, the progesterone concentrations were 0.273-, 1.62-, 0.392- and 2.06-fold, respectively.

The graph is presented in Figure 6.

## Discussion

In this study, we prepared endometrial sheets from rat endometrial tissue using cell sheet engineering and layered sheets, to create a novel hormone-responsive three-dimensional endometrial-like tissue with a thickness that allows observation of fertilized egg implantation and invasion. The 3D tissue was cultured in vitro at different progesterone concentrations and the histological and morphological changes of decidualization were observed to determine the appropriate local progesterone concentration for implantation of the fertilized egg.

Several in vitro embryo implantation assays have been reported to mimic endometrial tissue and embryos using cell lines and primary human cells [21]. Another study used primary endometrial cells or immortalized endometrial cells as endometrial tissue and fertilized eggs or immortalized trophoblast cells as fertilized eggs [7]. In these experimental systems, the constructed endometrial tissue was a two-dimensional culture, it was not sufficiently thick to enable the complete invasion and infiltration of the embryo. Furthermore, using cell lines may cause the endometrium to grow in a tumor-like fashion and may cause changes different from the normal endometrium when the fertilized egg is implanted. In some studies using 3D tissue with gel, fertilized egg invasion may be possible because 3D endometrial tissue is constructed; however, because the cell-cell distance is long and cell-to-cell adhesion is weak in gel-mixed tissue, it is inadequate as an experimental system to mimic the endometrium in vivo.

Depending on the size of the fertilized egg, it is necessary to provide tissues thicker than the diameter of the fertilized egg to observe implantation and infiltration. These endometrial tissue should have tight cell-to-cell interactions and be approximately 100 to 200 µm thick. In earlier studies, 3D endometrial tissue was created from primary rat tissue and it has been reported that when the normal endometrium was removed and replaced with 3D tissue, the fertilized egg implanted and grew to a fetus. This suggested that 3D endometrial tissue contribute to the regeneration of endometrial tissue in vivo [17-18]. Since this previous study indicated that the 3D tissue can regenerate endometrial tissue with normal function to conceive in vivo, the 3D tissue potentially possesses normal endometrial function in vitro. In this study, the cell sheet technology was used to create the 3D endometrial tissue without gel or other extraneous elements (Figure 1); therefore, cell-to-cell adhesion more closely resembled that of normal tissue, thereby facilitating the observation of tissue closer to that in a living body (Figure 2). In addition, 3D endometrial tissue was constructed with a thickness of 100 to 200 μm. Therefore, the sequential steps in the process of fertilized egg implantation may be observed in a near-physiological environment.

When cell sheets are stacked beyond a certain level, oxygen and nutrients become limiting as they are no longer supplied by diffusion alone, and the removal of waste products is limited by changing the culture medium. Even if short-term culture (a few days) is possible, it is difficult to maintain thick tissues for a long period without deformation or other changes.

The endometrial cell sheets in this study, prepared using the same method as those in these studies , showed cellular arrangements similar to those of normal endometrial tissue, and some endometrial glands were observed. In addition, progesterone receptors were detected, indicating hormone sensitivity (Figures 2, 4) [17-18].

It is established that the endometrial tissue requires a hormonal dedifferentiation reaction for embryo implantation and placenta formation, resulting in an increase in thickness and an increase in the number of endometrial glands. The number and ratio of endometrial glands can be affected by progesterone concentration. The endometrial glands are essential for pregnancy [22-23]. It has also been reported that increased progesterone concentrations increase the number and thickness of endometrial glands, leading to a higher pregnancy rate [24]. Another study showed that endometrial tissue cause “compaction” in thickness before and after decidualization occurs and this is expected to be a marker for predicting pregnancy rate; however, there are some negative reports regarding the prediction of pregnancy rate due to the change, and no conclusion has been reached as to whether or not, it is effective [25-26]. In this study, 3D endometrial tissues were cultured in four progesterone concentration groups and observed for desmoplasia. The P-4 group had the progesterone concentration required for culturing endometrial cell sheets and was used as a control. Referring to the progesterone concentration of fertilized eggs in culture, we defined the progesterone concentration in the local endometrium after ovulation as low concentration (P-200), medium concentration (P-1000), and high concentration (P-2000) groups [27]. It was possible to maintain 3D tissue with the addition of all progesterone concentration groups (Figure 3).

The thickness of the tissue was considered to be proportional to the progesterone concentration; however, the low-concentration group was thicker than the control group, whereas the medium- and high-concentration groups were thinner. This suggests that endometrial compaction did not occur in the low-concentration group but occurred in the medium- and high-concentration groups. Although no statistically significant difference was observed, the median and mean values of the high-concentration group were lower than those of the medium-concentration group, suggesting that compaction may be more advanced. The number of endometrial glands and area ratio in the tissues were higher in the low- and high-concentration groups than in the control group, and no significant differences were observed between the two groups. In contrast, the number and proportion of endometrial glands were low in the medium concentration group, suggesting that their development may have been suppressed. Although the number and ratio of endocrine glands would depend on progesterone concentration, the absence of significant differences between low and high concentrations in this study suggests that they may develop to some extent if the concentration is above a certain level. However, why the endometrial glands did not develop in the medium-concentration group remains a question for further study.

The gene expression of IGFBP-1, a marker of decidualization, increased, confirming that decidualization could be induced in all progesterone concentration groups. IGFBP-1 is a marker of decidualization, and it is believed that the higher its expression level, the more decidualization progresses. In this study, the number of samples was insufficient for statistical analysis. However, compared to the qPCR control, the expression was low in the control and medium concentration groups, whereas it was high in the low and high concentration groups. Gene expression levels also suggested that decidualization might have progressed in the low- and high-concentration groups, which was consistent with the number and ratio of endometrial glands. Some studies have reported that low blood progesterone concentrations on the date of embryo transplantation are associated with a low pregnancy rate , whereas high blood concentrations are associated with decreased pregnancy rates [28-29]. However, progesterone concentrations in local endometrial tissues do not correlate with serum concentrations [30]. This study suggests that there may not necessarily be a proportional relationship between the local endometrial progesterone concentration and decidualization.

The number of endometrial glands increased in the high concentration group, and compaction occurred, suggesting that among all concentration groups. These results suggest that an “appropriate local progesterone concentration” exists for good decidualization, which increases the pregnancy rate. Local progesterone concentration is important for “appropriate decidualization”, and our findings indicate that appropriate progesterone concentration should be controlled for “appropriate decidualization.” Moreover, new methods for measuring local hormonal concentrations may be needed to evaluate endometrial decidualization.

There are several limitations to this study. Since rat cells were used in this study, species differences from human cells must be considered. Other factors such as estrogen, insulin-like growth factor-1, and transforming growth factor-beta, which are necessary during fertilized egg implantation, were not added or studied. In addition, progesterone concentrations have been studied only in four groups, and there are no studies of concentrations above 2000 ng/ml. Moreover, investigator bias includes sample preparation, staining technique, and choice of stains and antibodies.

## Conclusions

In this study, we have developed a novel hormone-responsive 3D endometrial tissue model that can be used to study decidualization and embryo implantation. Induction of decidualization was observed with the addition of different concentrations of progesterone to the 3D endometrial tissue. The high concentration group with increased endometrial glands and compaction may meet the endometrial conditions that most effectively increase pregnancy rates; this can be a potential new approach for improving recurrent implantation failure. Construction of hormone-responsive 3D endometrial tissues has not been previously reported, this method suggests the best way to mimic the in-vivo endometrial environment to date. In the future, it is necessary to examine how decidualization is changed by adding higher progesterone concentrations. We plan to confirm what is “appropriate decidualization” by actually transplanting fertilized eggs and to check for changes in progesterone and estrogen receptor expression and immune tolerance. This method could enable the development of new testing and treatment methods to enhance implantation and pregnancy rates by identifying optimal conditions for decidualization and embryo implantation.
